# Prognostic significance of quantitative EBV biomarkers in extranodal NK/T-cell lymphoma: a meta-analysis of EBV DNA load and EBER-positive cell proportion

**DOI:** 10.1186/s13027-026-00752-9

**Published:** 2026-04-01

**Authors:** Xue song Wang, Di Liu, Xia Zhu, Nan Shi, Wei Xia, Anzhou Tang

**Affiliations:** 1 Department of Otorhinolaryngology Head and Neck Surgery, The First Affiliated Hospital of Guangxi Medical University, Nanning, Guangxi 530000 China; 2 Key Laboratory of Early Prevention and Treatment for Regional High Frequency Tumor, Guangxi Medical University, Ministry of Education, Nanning, Guangxi 530000 China; 3 Department of Otorhinolaryngology Head and Neck Surgery, Binzhou Central Hospital, Binzhou, Shandong 251700 China

**Keywords:** Extranodal NK/T-cell lymphoma, Epstein-Barr virus DNA, EBER expression

## Abstract

**Objectives:**

Pre-treatment peripheral blood EBV DNA and tumor EBV-encoded small RNA (EBER) are recognized prognostic factors in Extranodal NK/T-Cell Lymphoma (ENKTL). However, their quantitative values and optimal positive thresholds remain inconsistent, hindering clinical application. This meta-analysis systematically assesses their prognostic value in ENKTL.

**Methods:**

Relevant studies from PubMed, Embase, Web of Science, and Cochrane Library were searched until 24 December, 2025. Eligible studies reported hazard ratios (HRs) for overall survival (OS) based on Pre-treatment EBV DNA levels or tumor EBER positivity. Subsequently, Pooled HRs with 95% confidence intervals (CIs) were calculated via random-effects models. Next, Subgroup analyses examined different blood compartments, EBV DNA cut-offs, and study design. Additionally, Heterogeneity (I²) and publication bias (Egger’s test) were assessed.

**Results:**

In total, 17 studies from 15 articles were enrolled in this meta-analysis, including 15 on EBV DNA and 2 on EBER. These studies demonstrated that elevated pre-treatment EBV DNA level (HR = 3.04, 95% CI 1.97–4.69; *P* < 0.001, I^2^ = 38%) and high tumor EBER positivity thresholds of ≥ 75% (HR = 5.53, 95%CI2.21-14.38; *P* < 0.001, I^2^ = 0%) independently predicted reduced survival. Moreover, Stratification by EBV DNA cut-off (500 copies/ML) showed significantly higher mortality risk in the ≥ 500 group (HR = 2.82, 95% CI 1.84–4.31; *P* < 0.001) compared to the < 500 group (HR = 2.66, 95% CI 0.56–12.72; *P* = 0.22). Furthermore, Egger’s test suggested no publication bias(*P* = 0.111). Finally, Sensitivity analyses suggested the robustness and credibility of our results.

**Conclusions:**

High pre-treatment EBV DNA load (cut-off of ≥ 500 copies/mL) and high EBER-Positive Cell Proportion (≥ 75%) predict poor survival in ENKTL. Those quantitative EBV Biomarkers may serve as practical biomarkers for risk stratification and guiding therapeutic decisions in this patient population.

**Supplementary Information:**

The online version contains supplementary material available at 10.1186/s13027-026-00752-9.

## Introduction

ENKTL is an aggressive, EBV-associated non-Hodgkin lymphoma with regional variation [1]—most common in Asia (28%), followed by South America (11–15%) and the West (8%) [2–4]. No standard treatment exists, and despite combined chemotherapy, radiotherapy, and targeted therapies, advanced cases have a poor 5-year survival (< 35%) [5]. Thus, accurate prognostic tools are urgently needed to guide treatment.

Currently, prognostic models used in clinical practice include the International Prognostic Index (IPI), Korean Prognostic Index (KPI), Prognostic Index for Natural Killer Lymphoma (PINK), Prognostic Index for Natural Killer Lymphoma Incorporating EBV DNA (PINK-E), Nomogram-Revised Risk Index (NRI), and the SEC (SUV max-EBV DNA-ctDNA) nomogram, etc [6–10]. Notably, as traditional models like IPI and KPI do not fully incorporate biomarkers such as EBV DNA in peripheral blood, which can reflect the heterogeneity of ENKTL itself, PINK-E and the SEC nomogram integrating EBV DNA show better Prognostic stratification. These two indices demonstrate EBV-related biomarkers’ clinical significance.

For a long time, Pre-treatment EBV DNA quantification is a strong predictor of therapeutic responsiveness and survival outcomes in ENKTL, reflecting tumor burden [11, 12]. However, its role as an independent prognostic marker remains debated due to uncertainties in blood compartment choice [13, 14], EBV DNA cut-offs [15], and study design [16, 17].

Moreover, EBER in situ hybridization is commonly used, but lacks quantitative assessment of EBV-infected cells [18]. Studies on its prognostic value in ENKTL are inconsistent while some show no significant association with ENKTL outcomes [19], others report conflicting conclusions [20]. This may suggest that the determination of the EBER positivity threshold as a potential prognostic factor is still debated and remains controversial.

A previous meta-analysis suggested that EBV DNA levels in different blood compartments can indicate poor prognosis, particularly, plasma samples exhibited lower heterogeneity [21]. Moreover, when we chose the EBV DNA cut-off value as 0 copies/ML, it is hard to effectively assess the tumor’s prognosis [22]. Due to the small size of included studies, existing meta-analyses fail to systematically explore the impact of different cut-off values on tumor prognosis. Therefore, based on the mentioned meta-analysis. We aim to investigate the prognostic value of pre-treatment EBV DNA load and tumor EBER positivity in ENKTL.

## Methods

This study adhered to the Preferred Reporting Items for Systematic Reviews and Meta-Analyses (PRISMA) guidelines. The protocol was prospectively registered with PROSPERO (ID: CRD4202458009).

### Search strategy

Four electronic databases as follows were used: PubMed, Embase, Web of Science, and Cochrane Library by 24st December, 2025. We adopted a retrieval strategy that combines key words with free words. The keywords included “Lymphoma, Extranodal NK-T-Cell”, “Herpesvirus 4, Human”, and “prognosis”. The key terms and free words are interrelated through the Boolean logical operator “OR”, while the following key terms “Lymphoma, Extranodal NK-T-Cell”, “Herpesvirus 4, Human”, and “prognosis” are combined through the Boolean logical operator “AND” for the search. More details for searching are demonstrated in the supplementary materials. The detailed search strategy can be found in Supplementary Table S1 (PRISMA 2020 Checklist) and Table S2 (Detailed search strategies).

### Inclusion and exclusion criteria

Inclusion criteria required that all selected studies:

1. studies on newly diagnosed ENKTL patients based on histopathology;
2. Studies evaluating EBV Infection and overall survival (OS);
3. Directly reported HR and 95% CI (preferred) OR provide Kaplan-Meier survival curves allowing HR estimation via digitization tools and the methods illustrated by Tierney et al. [23].

Exclusion criteria were as follows:

1. duplicate articles;
2. conference and meeting abstracts;
3. reviews, meta-analyses, case reports, letters, short communications, and editorials;
4. animal or cell experiments;
5. inconsistent with the study: mismatched diagnosis, unclear newly diagnosis of ENKTL, lack of information on pre-treatment EBV DNA, relapse/refractory ENKTL;
6. studies do not provide HR values or HR values cannot be extracted from survival curves;
7. Lack of standardized blood compartments, EBV DNA cut-offs, or EBER positivity thresholds.

### Data curation

We independently assessed each included report and extracted relevant data. Any discrepancies were resolved through group discussions. Extracted information as follows: the first author’s name, year of publication, Country, sample type, number of EBV-positive and negative patients, cut-off values of Pre-treatment EBV DNA, EBER positivity thresholds, type of research, and whether to perform multivariate analyses.

### Quality assessment of included studies

We respectively used a tool-the Newcastle-Ottawa Scale (NOS) to assess the quality of included studies. The NOS evaluates studies across three domains, with a maximum of 9 points: Selection (up to 4 points), Comparability (up to 2 points) and outcome (up to 3 points) [24]. Reports with a score of ≥ 6 will be marked as high-quality. Any discrepancies were resolved through group discussion.

### Statistical analysis

Directly retrieving HR and 95% CI from the reported results is the most accurate method. However, if the study did not report the HR and 95% CI, these can be calculated by extracting survival rates from OS curves with a tool of Engauge Digitizer(Version 12.1; Mitchell, 2020). Statistical analyses of these included studies were conducted using software like Reviewer Manager 5.3 (Copenhagen: The Nordic Cochrane Centre; The Cochrane Collaboration, 2014) and Stata (Version 14.0; Stata Corp, College Station, TX, USA). Then, the heterogeneity was estimated by means of the χ2 heterogeneity statistic Q and then quantified using I^2^, a measurement that describes the percentage of total variation rather than chance [25]. An I^2^ value equal to 25%, 50% and 75% indicated low, moderate and high variation respectively. A fixed-effects model was applied when heterogeneity was insignificant, while a random-effects model was used when heterogeneity was significant [26].

To further explore the sources of heterogeneity, we conducted subgroup analyses based on Blood compartment, Cut-off, and Type of study. Additionally, publication bias was assessed by Egger’s test. The trim-and-fill method was applied for detecting and adjusting significant biases. *P* < 0.05 was considered statistically significant.

## Results

### Characteristics of included studies

**Fig. 1:**
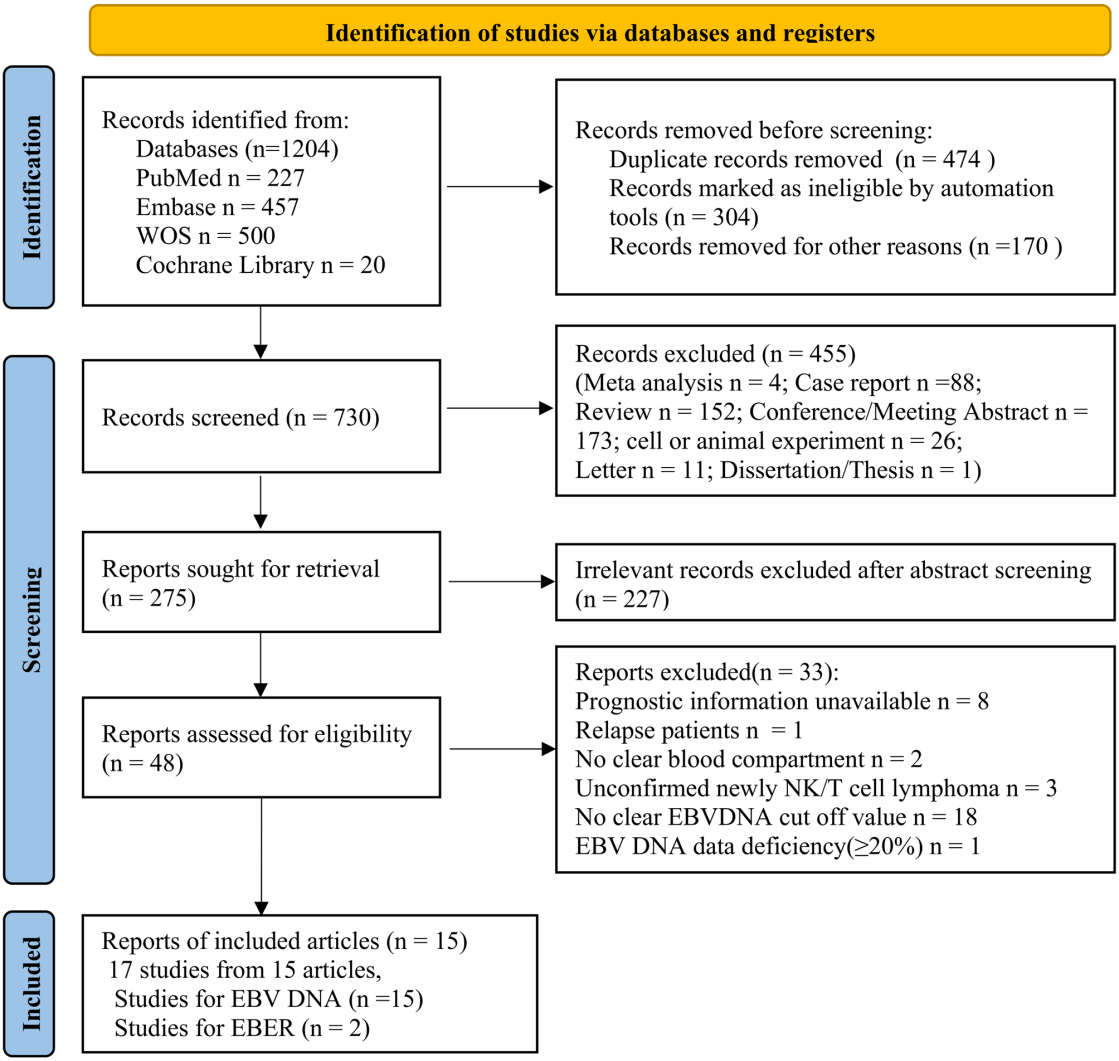
PRISMA flow diagram of literature search and selection. Abbreviations: PRISMA: Preferred Reporting Items for Systematic Reviews and Meta-Analyses. EBV: Epstein-Barr virus. EBER: Epstein-Barr virus-encoded small RNA (or Epstein-Barr virus-encoded small RNAs)

By 24st December, 2025, we retrieved a total of 1204 articles from PubMed, Embase, Web of Science, and Cochrane Library. Among these studies, 474 duplicate papers were excluded, and 48 full-text articles were selected based on abstract screening. With a further evaluation, 15 articles (involving 1232ENKTL patients) met the inclusion criteria and were included in the meta-analysis. Two of the selected 15 studies investigated in different blood compartments. Each study should be considered independently. Therefore, 17 studies from 15 articles were enrolled in this meta-analysis, including 15 on EBV DNA and 2 on EBER (Fig. 1). The characteristics of the included studies are sorted in Table 1, and these papers are evaluated using the NOS tool (Table 2).

**Table 1:** Methodological and demographic profiles of 15 included articles (17 independent studies)

Study	Year	Country	Sample type	Total patients	Pre-treatment EBV DNA+/-	EBVDNA Cut-off (copies/mL)	EBER positivity	Study type	Multivariate analysis	Outcomes
Kim et al. [27]	2009	South Korea	Whole Blood	47	13/34	63.61*10^3^	NA	Retrospective	NO	OS
Suzuki et al. [28]	2011	Japan	Tumor tissue	32	NA	NA	75%	Prospective	YES	OS
Ito (1) et al. [29]	2012	Japan	Whole Blood	26	22/4	10^5^	NA	Prospective	YES	OS
Ito (2) et al.[29]	2012	Japan	Tumor tissue	26	NA	NA	75%	Prospective	NO	OS
Wang et al. [30]	2012	China	Plasma	69	58/11	500	NA	Prospective	NO	OS
Kim et al. [31]	2015	South Korea	Whole Blood	27	17/10	6.0*10^4^	NA	Prospective	YES	OS
Wang et al. [32]	2015	China	Plasma	68	43/25	0	NA	Prospective	NO	OS
Chen et al. [33]	2017	China	PBMC	45	33/12	5,000	NA	Retrospective	YES	OS
Wei et al. [34]	2017	China	Serum	35	10/25	6.1*10^7^	NA	Retrospective	NO	OS
Wang et al. [35]	2019	China	Plasma	99	77/22	0	NA	Retrospective	YES	OS
Zhao et al. [36]	2019	China	Plasma	55	25/30	500	NA	Prospective	NO	OS
Ha (1) et al. [37]	2020	South Korea	Whole Blood	60	37/23	457	NA	Prospective	YES	OS
Ha (2) et al. [37]	2020	South Korea	Plasma	60	23/37	457	NA	Prospective	YES	OS
Mao et al. [38]	2021	China	Whole Blood	93	50/43	5000	NA	Retrospective	NO	OS
Sun et al. [39]	2022	China	Plasma	391	166/225	502	NA	Retrospective	YES	OS
Wang et al. [40]	2023	China	Plasma	134	75/59	500	NA	Retrospective	YES	OS
Chan et al. [41]	2025	China	Plasma	51	24/27	3250	NA	Retrospective	YES	OS

Abbreviations: EBV, Epstein–Barr virus; EBER, Epstein-Barr virus-encoded small RNA; PBMC, peripheral blood mononuclear cells; OS, overall survival

**Table 2:** Risk of bias assessment in included studies using the newcastle-ottawa scale (NOS)

Included studies	Selection(0–4)				Comparability(0–2)		Outcome (0–3)			NOS score
	REC	SENC	AE	DO	SC	AF	AO	FU	AFU	
Kim et al. 2009 [27]	1	1	1	1	0	0	1	1	1	7
Suzuki et al. 2011 [28]	1	1	1	1	0	0	1	1	1	7
Ito et al. 2012 [29]	1	1	1	1	0	0	1	1	1	7
Wang et al. 2012 [30]	1	1	1	1	1	0	1	1	1	8
Kim et al. 2015 [31]	1	1	1	1	0	0	1	1	1	7
Wang et al. 2015 [32]	1	1	1	1	1	0	1	1	1	8
Chen et al. 2017 [33]	1	1	1	1	1	0	1	1	1	8
Wei et al. 2017 [34]	1	1	1	1	0	0	1	1	1	7
Wang et al. 2019 [35]	1	1	1	1	1	0	1	1	1	8
Zhao et al. 2019 [36]	1	1	1	1	0	0	1	1	1	7
Ha et al. 2020 [37]	1	1	1	1	0	0	1	1	1	7
Mao *et a*^]^ 2021 [38]	1	1	1	1	0	0	1	1	1	7
Sun et al. 2022 [39]	1	1	1	1	0	0	1	1	1	7
Wang et al. 2023 [40]	1	1	1	1	0	0	1	1	1	7
Chan et al. 2025 [41]	1	1	1	1	0	0	1	1	1	7

Note: High-quality: 7–9 stars (rigorous methodology with minimal risk of bias); Moderate-quality: 4–6 stars (methodological limitations requiring cautious interpretation)༛ Low-quality: <4 stars (substantial validitconcerns, excluded from primary analysis)

Abbreviations: AE, ascertainment of exposure; AF, study controls for any additional factors (chemoradiotherapy, curative resection); AFU, adequacy of follow-up of

cohorts; AO, assessment of outcome; DO, demonstration that outcome of interest was not present at start of study; FU, follow-up long enough for outcomes to occur;

REC, representativeness of the exposed cohort; SC, study controls for age, sex; SNEC, selection of the non-exposed cohort

### Prognostic impact of pre-treatment EBV DNA level and EBER positivity thresholds

**Fig. 2:**
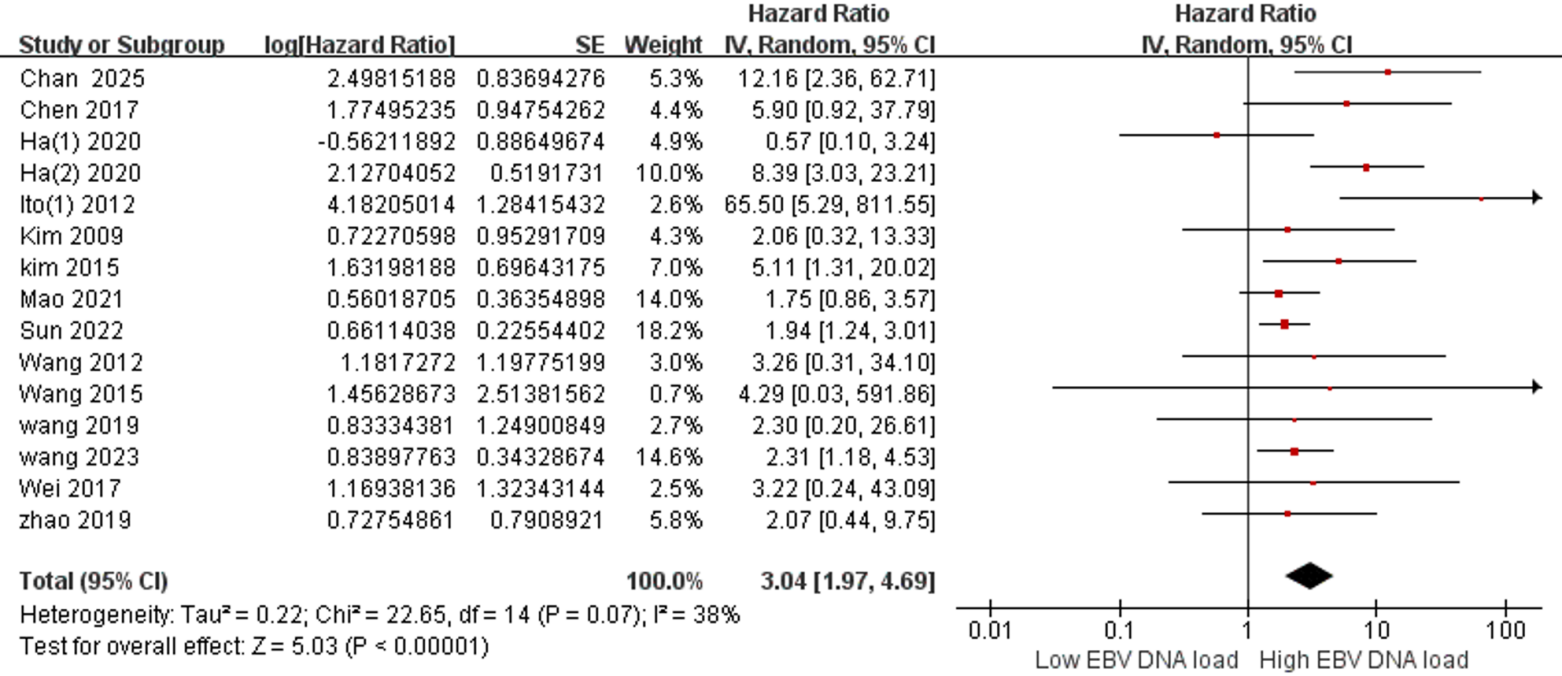
Estimated hazard ratios for OS in all pre-treatment EBV DNA. Abbreviations: SE: Standard error; IV: Inverse variance; CI: Confidence interval; OS: Overall survival

As demonstrated in Fig. 2, for included 15 studies about pre-treatment EBV DNA in peripheral blood, a moderate heterogeneity is indicated by the results of Q and I^2^ test (Q = 22.65, *P* = 0.07 < 0.1, I^2^ = 38%), so a random-effects model was chosen for the meta-analysis. The combined effect size reaches HR = 3.04 and 95% CI (1.97–4.69), which was statistically significant (Z = 5.03, *P* < 0.001).

**Fig. 3:**
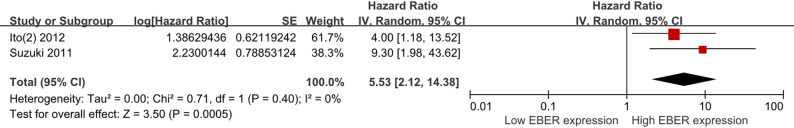
Estimated hazard ratios for OS in tumor EBER expression

As illustrated in Fig. 3, for the included 2 articles with 2 studies about EBER Expression in tumor tissues, a lower heterogeneity is indicated by the results of Q test and I ^2^ test (*P* = 0.40>0.1, I^2^ = 0%), so a fixed-effects model was chosen for the meta-analysis. The combined effect size reaches HR = 5.53 and 95% CI (2.12–14.38), which was statistically significant (Z = 3.50, *P* < 0.001).

### Subgroup analysis

Subgroup analyses further examined the relationship between pre-treatment EBV DNA levels and OS to address inconsistencies within the studies (Table 3).

**Table 3:** Subgroup analysis of included studies on pre-treatment EBV DNA and OS

Subgroups analysis	Studies	HR (95%CI)	Z	*P*	Model	Test of heterogeneity		
						X^2^	*P*	I^2^(%)
**Overall**	15	3.04(1.97–4.69)	5.03	0.000	Random	22.65	0.07	38
**Blood compartment**								
Whole Blood /PBMC	6	3.17(1.20–8.38)	2.33	0.02	Random	12.12	0.03	59
Plasma/Serum	9	3.00(1.89–4.74)	4.68	0.000	Random	10.50	0.23	24
**Cut-off(500copies/ml)**								
<500	4	2.66(0.56–12.72)	1.23	0.22	Random	7.06	0.07	58
≥ 500	11	2.82(1.84–4.31)	4.77	0.000	Random	14.31	0.16	30
**Type of study**								
Retrospective	8	2.20(1.61–2.99)	5.01	0.000	Random	6.08	0.53	0
Prospective	7	4.26(1.60-11.31)	2.91	0.004	Random	12.29	0.06	51

### Different blood compartments

**Fig. 4:**
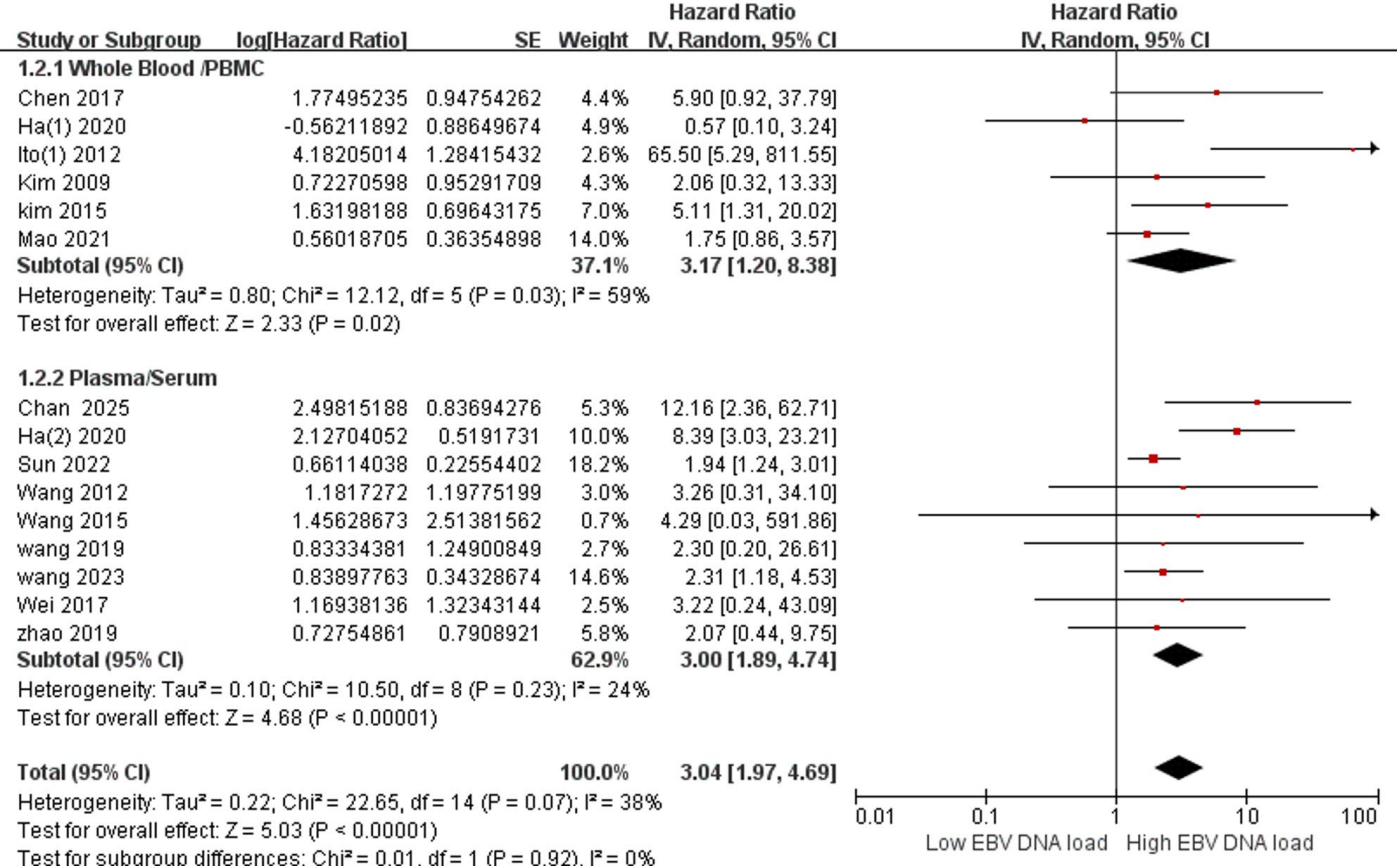
Estimated hazard ratios for OS in Plasma/Serum group and Whole Blood/PBMC group

Data were stratified by sample type as follows: the whole blood/PBMC (peripheral blood mononuclear cells) group (HR = 3.17, 95% CI 1.20–8.38; I² =59%) and the plasma/serum group (HR = 3.00, 95% CI 1.89–4.74; I² =24%), with detailed results shown in Fig. 4. From the results, we can detect that both plasma and whole blood groups can successfully predict tumor prognosis.

### Pre-treatment EBVDNA cut-off values

**Fig. 5:**
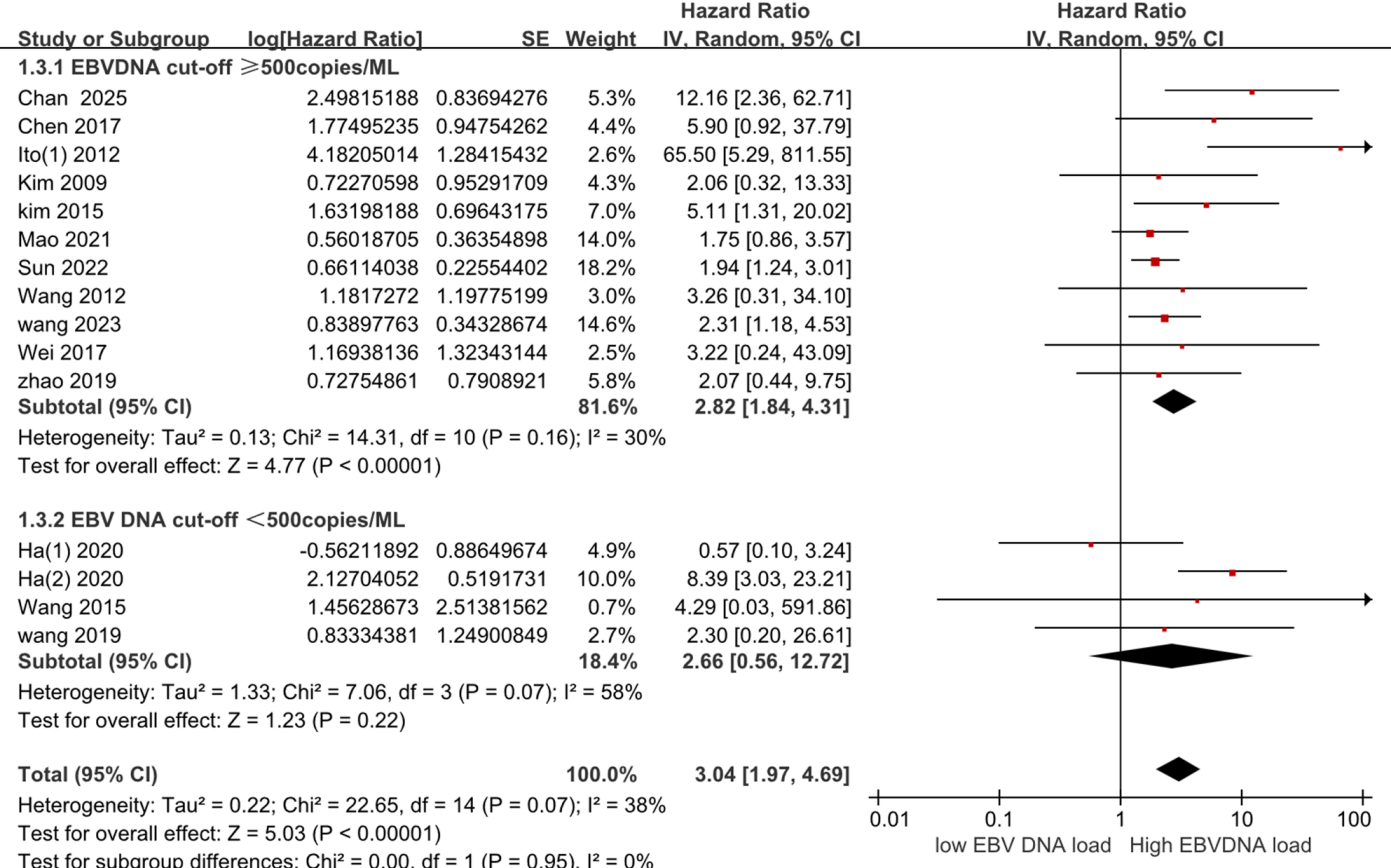
Estimated hazard ratios for OS in EBVDNA Cut-off < 500copies/ml and Cut-off ≥  500copies/ml group

The data were stratified as follows: the ≥ 500 copies/ML group (HR = 2.82, 95% CI 1.84–4.31; I² = 30%; *P* < 0.001) and the < 500 copies/ML group (HR = 2.66, 95% CI 0.56–12.72; I² = 58%; *P* = 0.22), with further details presented in Fig. 5.The results showed that EBV DNA levels below this threshold did not show a significant increase in risk, whereas the ≥ 500 copies/ML group had significantly worse outcomes.

### Prospective and retrospective studies

**Fig. 6:**
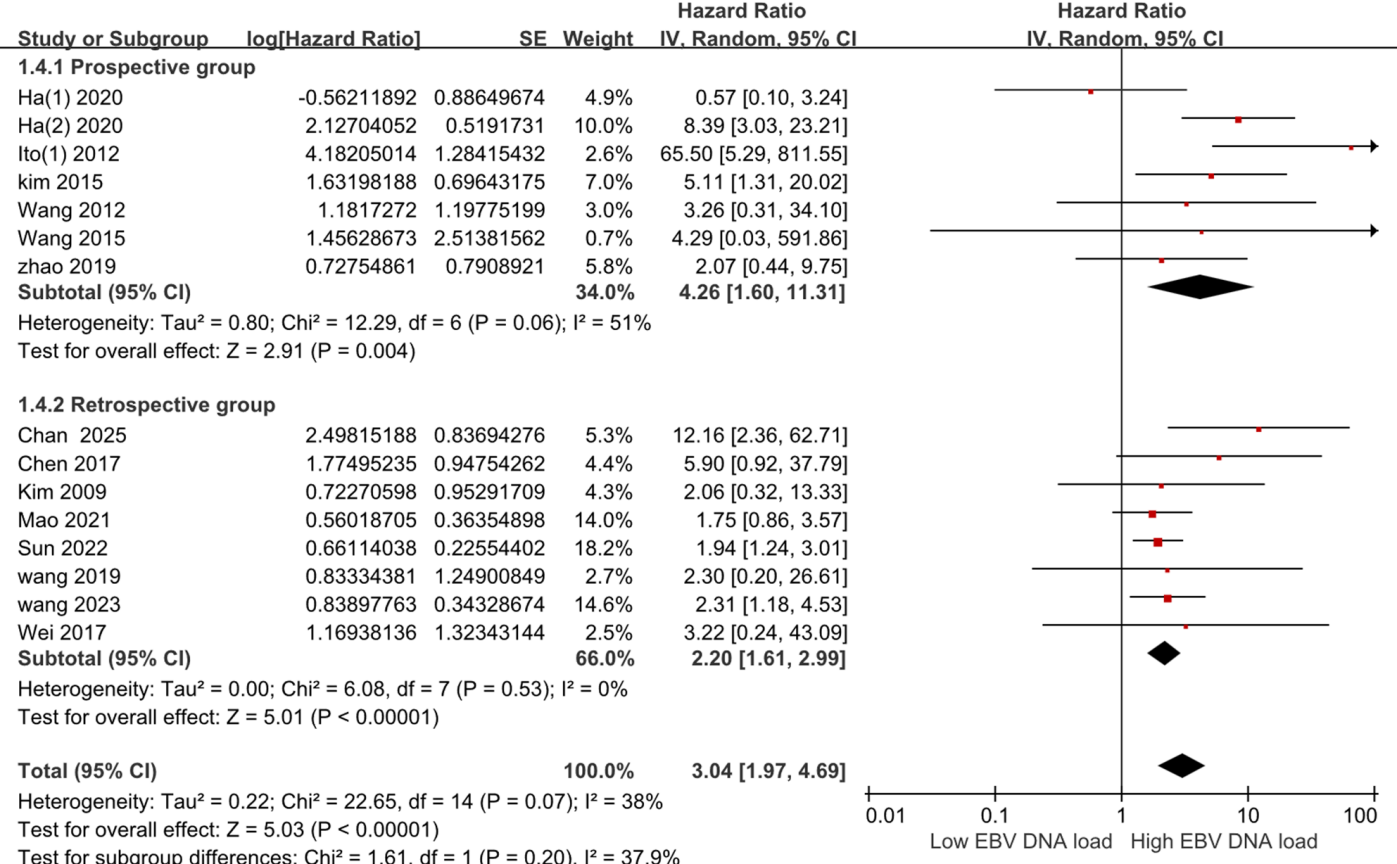
Estimated hazard ratios for OS in retrospective and prospective group

The prospective cohort exhibited a significantly elevated risk (HR = 4.26, 95% CI 1.60–11.31; I² = 51%) versus the retrospective cohort (HR = 2.20, 95% CI 1.61–2.99; I² = 0%), as detailed in Fig. 6. From the results, we can detect that both retrospective and prospective studies can successfully predict tumor prognosis.

### Publication bias and sensitivity analysis

**Fig. 7:**
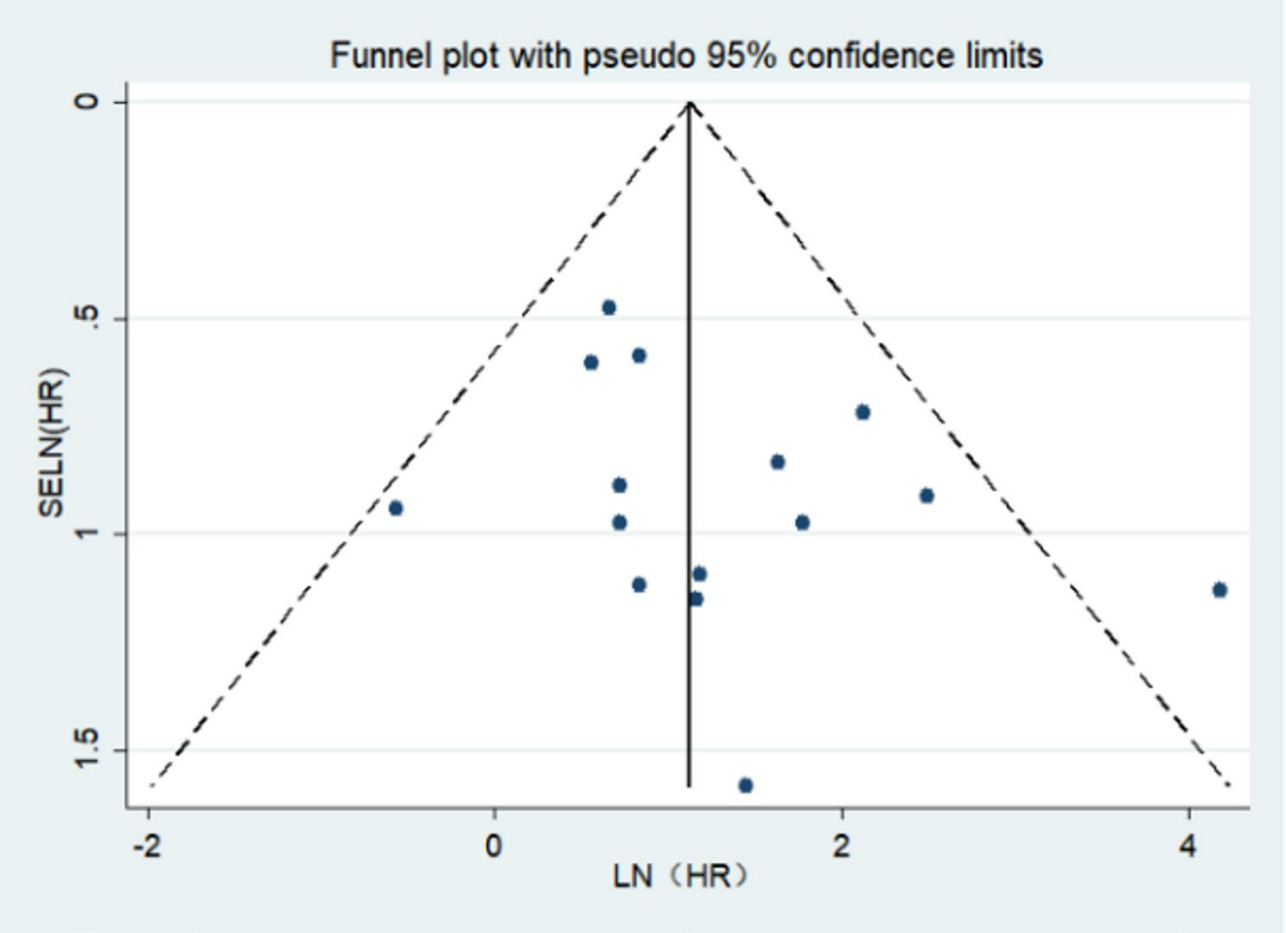
Funnel plots of publication bias on the association between pre-treatment EBV DNA and OS

**Fig. 8:**
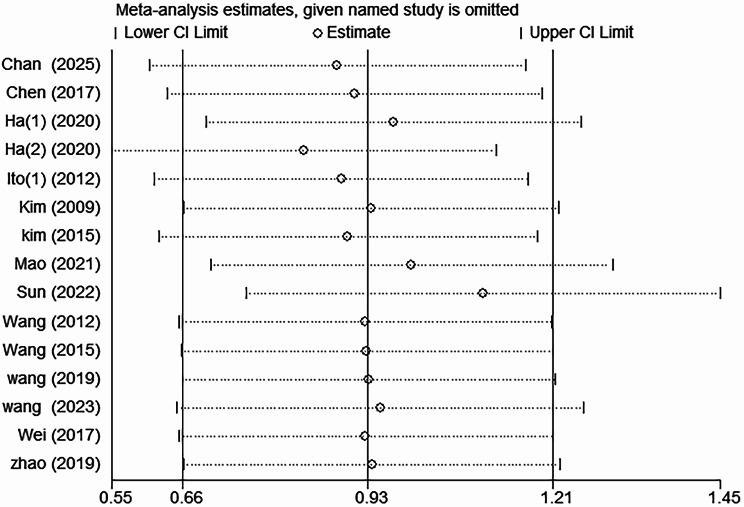
Sensitivity analysis on the association between pre-treatment EBV DNA and OS

From the funnel plot demonstrates no obvious bias (Fig. 7). Then, a further Egger’s bias test yielded a *P* = 0.111>0.05, which indicates that the publication bias observed in this meta-analysis is no bias. therefore, the current meta-analysis result is relatively stable. Moreover, we performed a sensitivity analysis by randomly deleting any individual study. Results suggested that our outcome was not significantly affected, which demonstrated that the result of the random effects model was stable and statistically reliable (more details in Fig. 8).

## Discussion

In this meta-analysis, pre-treatment peripheral blood EBV DNA positivity is an adverse prognostic factor for OS. This result is generally consistent with previous meta-analysis [21, 22]. However, moderate heterogeneity exists in the combined results. Therefore, further investigation is required to explore the sources of heterogeneity, focusing on the following three factors: blood compartment, positive cut-off values, and study type for their impact on the prognosis of newly diagnosed ENKTL patients.

Firstly, the subgroup analysis based on different blood compartment sources showed that pre-treatment positive EBV DNA indicates a poor prognosis in both plasma and whole blood groups. However, the heterogeneity in the plasma group is significantly lower than that in the whole blood group. This difference may be related to the EBV latent infection characteristics of memory B cells in whole blood. The memory B cells in whole blood can periodically release EBV genomic fragments into the blood, reducing the correlation between EBV DNA load and tumor burden [27]. In contrast, the EBV DNA in plasma primarily comes from free viral DNA released from tumor cell lysis, so the test of this group can reflect tumor burden more directly [28]. The results of this subgroup analysis are consistent with previous meta-analysis findings [21, 22]. Although EBVDNA positivity in different compartment sources can predict ENKTL prognosis, the plasma group has greater stability in the tumor prognosis evaluation.

Secondly, this meta-analysis underscores the need for exploratory identification of positive EBV DNA cut-off values in ENKTL prognosis. A previously meta-analysis showed EBV DNA positivity at 0 copies/ML cannot independently predict ENKTL prognosis [22]. Currently, most studies used 500 copies/ML as EBV DNA positivity, though its role as an independent prognostic factor remains debated [29–31]. Therefore, we plan to select 500 copies/ML as the positive cut-off value for pooled effect size analysis. The analysis demonstrated that EBV DNA levels below the predefined threshold were not associated with a significant increase in risk (high heterogeneity observed), while patients with EBV DNA levels ≥ 500 copies/ML exhibited significantly poorer clinical outcomes with lower heterogeneity. This observed phenomenon may be explained by the fact that low-level EBV DNA likely originates from EBV latent infections, which exhibit weaker associations with disease activity, thereby limiting their prognostic utility [28, 32]. In contrast, the unequivocally poor outcomes in the high-threshold group reflect the robustness of elevated EBV DNA as a biomarker of tumor burden [33, 34]. These findings underscore the need for multicenter validation of the 500 copies/ML threshold to confirm its independence.

Thirdly, from the subgroup analysis based on study type, we can find that both retrospective and prospective studies can predict the poor prognosis of ENKTL patients with positive plasma EBV DNA. In the retrospective study group, there was no heterogeneity, while the prospective study group showed moderate heterogeneity. Given the rarity of ENKTL, most researchers adopted the type of retrospective study whose sample sizes are easier to be obtained but may involve data deficiencies [31, 32, 34–39]. On the other hand, some researchers use prospective studies, which usually have more complete follow-up data but smaller sample sizes, potentially resulting in some variations in the findings [30, 33–41–28]. This meta-analysis is consistent with previous results [21, 22]. Therefore, the retrospective study group has studies without heterogeneity, and its combined HR value was more stable. In a previous randomized controlled trial involving 80 patients, the authors investigated how EBV-DNA status modifies the treatment effect of the DDGP regimen compared with the SMILE regimen [42]. Among EBV-DNA negative patients, the DDGP regimen demonstrated a significant advantage over the SMILE regimen; however, this difference was not statistically significant in EBV-DNA positive patients. These findings suggest that EBV-DNA status may serve as a predictive biomarker for differential response to chemotherapy regimens. However, given the prospective studies having more convincing results, larger-scale, multi-center prospective cohort studies are still required for verification.

This meta-analysis also identified a novel finding: high EBER expression (≥ 75% positive tumor cells) is significantly associated with inferior OS in ENKTL. Although EBER positivity is widely used diagnostically, it is typically assessed qualitatively, not quantitatively [18]. Despite the WHO recommending an EBER positivity threshold of ≥ 80% [43], clinical standardization remains difficult due to tissue processing variability, observer differences, and technical constraints. Notably, only two studies in our analysis quantitatively evaluated EBER, both limited to univariate analysis [33, 44]. This highlights the preliminary nature of our finding and underscores the need for large, multicenter studies to validate its prognostic value.

### Limitations

Some limitations exist in this meta-analysis as follows: (1) This study is based solely on published literature, so individual patient survival data could not be directly obtained. (2) Only pre-treatment EBV DNA information was included, and dynamic changes in EBV DNA were considered. (3) Publication bias could not be evaluated given the two-study limitation for EBER and OS. (4) Extracting hazard ratios (HRs) from published Kaplan-Meier curves, some degree of error may occur during the data extraction process. (5) This study researches various cut-off values based on retrospective studies and small sample prospective studies. That means further large-scale, multi-center prospective studies are still needed for validation. (6) Since this study primarily focused on the impact of EBV infection on the prognosis of ENKTL, numerous other prognostic markers for ENKTL warrant further investigation, such as P53 mutation status, CD30 expression, and mutations in STAT3, JAK3, BCOR, and DDX3X.

## Conclusions

In conclusion, High pre-treatment EBV DNA load (cut-off of ≥ 500 copies/mL) and high EBER-Positive Cell Proportion (≥ 75%) predict poor survival in ENKTL. Those Quantitative EBV Biomarkers may serve as practical biomarkers for risk stratification and guiding therapeutic decisions in this patient population.

## Supplementary Information

Below is the link to the electronic supplementary material.

Supplementary Material 1

## Data Availability

The raw data supporting the conclusions of this article will be made available by the authors, without undue reservation.

## References

[1] Kwong YL. Natural killer-cell malignancies: diagnosis and treatment. Leukemia. 2005;19(12):2186–94. 10.1038/sj.leu.2403955. 16179910 10.1038/sj.leu.2403955

[2] Chen JJ, Tokumori FC, Del Guzzo C, Kim J, Ruan J. Update on T-Cell Lymphoma Epidemiology. Curr Hematol Malig Rep. 2024;19(3):93–103. 10.1007/s11899-024-00727-w. 38451372 10.1007/s11899-024-00727-w

[3] Yoon SE, Song Y, Kim SJ, et al. Comprehensive analysis of peripheral T-cell and natural killer/T-cell lymphoma in Asian patients: A multinational, multicenter, prospective registry study in Asia. Lancet Reg Health West Pac. 2021;22(10):100126. 10.1016/j.lanwpc.2021.100126 PMC8315366 34327343

[4] Malpica L, Idrobo H, Pavlovsky A, et al. Epidemiology, clinical features, and outcomes of peripheral T-cell lymphoma in Latin America: an international, retrospective, cohort study. Lancet Haematol. 2025;12(4):e258–68. 10.1016/S2352-3026(25)00011-0. 40056928 10.1016/S2352-3026(25)00011-0

[5] Wei YC, Qi F, Zheng BM, et al. Intensive therapy can improve long-term survival in newly diagnosed, advanced-stage extranodal NK/T-cell lymphoma: A multi-institutional, real-world study. Int J Cancer. 2023;153(9):1643–57. 10.1002/ijc.34672. 37539660 10.1002/ijc.34672

[6] Chim CS, Ma SY, Au WY, et al. Primary nasal natural killer cell lymphoma: long-term treatment outcome and relationship with the International Prognostic Index. Blood. 2004;103(1):216–21. 10.1182/blood-2003-05-1401. 12933580 10.1182/blood-2003-05-1401

[7] Lee J, Suh C, Park YH, et al. Extranodal natural killer T-cell lymphoma, nasal-type: a prognostic model from a retrospective multicenter study. J Clin Oncol. 2006;24(4):612–8. 10.1200/JCO.2005.04.1384. 16380410 10.1200/JCO.2005.04.1384

[8] Kim SJ, Yoon DH, Jaccard A, et al. A prognostic index for natural killer cell lymphoma after non-anthracycline-based treatment: a multicentre, retrospective analysis. Lancet Oncol. 2016;17(3):389–400. 10.1016/S1470-2045(15)00533-1. 26873565 10.1016/S1470-2045(15)00533-1

[9] Chen SY, Yang Y, Qi SN, et al. Validation of nomogram-revised risk index and comparison with other models for extranodal nasal-type NK/T-cell lymphoma in the modern chemotherapy era: indication for prognostication and clinical decision-making. Leukemia. 2021;35(1):130–42. 10.1038/s41375-020-0791-3. 32152465 10.1038/s41375-020-0791-3 PMC7787971

[10] Huang D, Li F, Lin S, et al. A novel prognostic nomogram based on imaging and molecular parameters for newly diagnosed extranodal natural killer/T-cell lymphoma patients. Haematologica. 2025;110(1):200–5. 10.3324/haematol.2024.285362. 39157873 10.3324/haematol.2024.285362 PMC11694128

[11] Kimura H, Kwong YL. EBV Viral Loads in Diagnosis, Monitoring, and Response Assessment. Front Oncol. 2019;9:62. 10.3389/fonc.2019.00062. 30809508 10.3389/fonc.2019.00062 PMC6379266

[12] Tse E, Zhao WL, Xiong J, Kwong YL. How we treat NK/T-cell lymphomas. J Hematol Oncol. 2022;15(1):74. 10.1186/s13045-022-01293-5. 35659326 10.1186/s13045-022-01293-5 PMC9164389

[13] Yan Z, Yao Z, Wang H, et al. Plasma EBV DNA and peripheral blood mononuclear cell EBV DNA have disparate clinical relevance in patients with extranodal NK/T-cell lymphoma. J Clin Virol. 2022;157:105320. 10.1016/j.jcv.2022.105320. 36240675 10.1016/j.jcv.2022.105320

[14] Zheng M, Bao Y, Wang J, et al. The superiority of Epstein-Barr virus DNA in plasma over in peripheral blood mononuclear cells for monitoring EBV-positive NK-cell lymphoproliferative diseases. Hematol Oncol. 2022;40(3):381–9. 10.1002/hon.2998. 35405763 10.1002/hon.2998

[15] Fryer JF, Heath AB, Wilkinson DE, et al. A collaborative study to establish the 1st WHO International Standard for Epstein-Barr virus for nucleic acid amplification techniques. Biologicals. 2016;44(5):423–33. 10.1016/j.biologicals.2016.04.010. 27461128 10.1016/j.biologicals.2016.04.010

[16] Kim JJ, Kim HY, Choi Z, et al. In-depth circulating tumor DNA sequencing for prognostication and monitoring in natural killer/T-cell lymphomas. Front Oncol. 2023;13:1109715. 10.3389/fonc.2023.1109715. 36845680 10.3389/fonc.2023.1109715 PMC9954142

[17] Hai T, Wu W, Ren K, Li N, Zou L. Prognostic significance of the systemic immune-inflammation index in patients with extranodal natural killer/T-cell lymphoma. Front Oncol. 2023;13:1273504. 10.3389/fonc.2023.1273504. 37909016 10.3389/fonc.2023.1273504 PMC10613892

[18] Tomomasa D, Tanita K, Hiruma Y, et al. Highly sensitive detection of Epstein-Barr virus-infected cells by EBER flow FISH. Int J Hematol. 2024;120(2):241–51. 10.1007/s12185-024-03786-0. 38700651 10.1007/s12185-024-03786-0

[19] Fu XR, Wan WJ, Sun ZC, et al. [Expression of CD7 and its correlation with prognosis in patients with NK/T-cell lymphoma]. Zhonghua Xue Ye Xue Za Zhi. 2020;41(11):921–6. 10.3760/cma.j.issn.0253-2727.2020.11.007. Chinese. 33333695 10.3760/cma.j.issn.0253-2727.2020.11.007 PMC7767813

[20] Song J, Kim JY, Kim S, Park Y. Utility of Epstein-Barr Viral Load in Blood for Diagnosing and Predicting Prognosis of Lymphoma: A Comparison with Epstein-Barr Virus-Encoded RNA in Situ Hybridization. J Mol Diagn. 2022;24(9):977–91. 10.1016/j.jmoldx.2022.06.002. 35718093 10.1016/j.jmoldx.2022.06.002

[21] Chen R, Wang C, Zhou Y, Wen B. Prognostic implications of circulating Epstein-Barr virus DNA for extranodal natural killer/T-cell lymphoma, nasal type: a meta-analysis. Cancer Manag Res. 2018;10:2183–92. 10.2147/CMAR.S162168. 30050327 10.2147/CMAR.S162168 PMC6056158

[22] Fei Q, Tian XK, Wu J, et al. Prognostic significance of Epstein-Barr virus DNA in NK/T-cell lymphoma: a meta-analysis. Onco Targets Ther. 2018;11:997–1004. 10.2147/OTT. 29520150 10.2147/OTT.S153942 PMC5833780

[23] Tierney JF, Stewart LA, Ghersi D, Burdett S, Sydes MR. Practical methods for incorporating summary time-to-event data into meta-analysis. Trials. 2007;8:16. 10.1186/1745-6215-8-16. 17555582 10.1186/1745-6215-8-16 PMC1920534

[24] Stang A. Critical evaluation of the Newcastle-Ottawa scale for the assessment of the quality of nonrandomized studies in meta-analyses. Eur J Epidemiol. 2010;25(9):603–5. 10.1007/s10654-010-9491-z. 20652370 10.1007/s10654-010-9491-z

[25] Higgins JP, Thompson SG. Quantifying heterogeneity in a meta-analysis. Stat Med. 2002;21(11):1539–58. 10.1002/sim.1186. 12111919 10.1002/sim.1186

[26] Higgins JP, Thompson SG, Deeks JJ, Altman DG. Measuring inconsistency in meta-analyses. BMJ. 2003;327(7414):557–60. 10.1136/bmj.327.7414.557. 12958120 10.1136/bmj.327.7414.557 PMC192859

[27] Allday MJ, Bazot Q, White RE. The EBNA3 Family: Two Oncoproteins and a Tumor Suppressor that Are Central to the Biology of EBV in B Cells. Curr Top Microbiol Immunol. 2015;391:61–117. 10.1007/978-3-319-22834-1_3. 26428372 10.1007/978-3-319-22834-1_3

[28] Ha JY, Cho H, Sung H, et al. Superiority of Epstein-Barr Virus DNA in the Plasma Over Whole Blood for Prognostication of Extranodal NK/T Cell Lymphoma. Front Oncol. 2020;10:594692. 10.3389/fonc.2020.594692. 33330083 10.3389/fonc.2020.594692 PMC7734249

[29] Wang ZY, Liu QF, Wang H, et al. Clinical implications of plasma Epstein-Barr virus DNA in early-stage extranodal nasal-type NK/T-cell lymphoma patients receiving primary radiotherapy. Blood. 2012;120(10):2003–10. 10.1182/blood-2012-06-435024. 22826562 10.1182/blood-2012-06-435024

[30] Zhao Q, Fan S, Chang Y, et al. Clinical efficacy of cisplatin, dexamethasone, gemcitabine and pegaspargase (DDGP) in the initial treatment of advanced stage (stage III-IV) extranodal NK/T-cell lymphoma, and its correlation with Epstein-Barr virus. Cancer Manag Res. 2019;11:3555–64. 10.2147/CMAR.S191929. 31118779 10.2147/CMAR.S191929 PMC6497975

[31] Wang T, Qiu Y, Shi L, et al. Dynamic Prediction of Survival for Sinonasal Extranodal Natural Killer/T-cell Lymphoma. Laryngoscope. 2023;133(7):1645–51. 10.1002/lary.30342. 37294046 10.1002/lary.30342

[32] Wang XX, Li PF, Bai B, et al. Differential clinical significance of pre-, interim-, and post-treatment plasma Epstein-Barr virus DNA load in NK/T-cell lymphoma treated with P-GEMOX protocol. Leuk Lymphoma. 2019;60(8):1917–25. 10.1080/10428194.2018.1563690. 30646796 10.1080/10428194.2018.1563690

[33] Ito Y, Kimura H, Maeda Y, et al. Pre-treatment EBV DNA copy number is predictive of response and toxicities to SMILE chemotherapy for extranodal NK/T-cell lymphoma, nasal type. Clin Cancer Res. 2012;18(15):4183–90. 10.1158/1078-0432. 22675173 10.1158/1078-0432.CCR-12-1064

[34] Chan CY, Lin TL, Kuo MC, et al. Prognostic impact of pre-treatment and post-treatment plasma Epstein-Barr virus DNA in peripheral T-cell lymphomas. Ann Med. 2025;57(1):2478315. 10.1080/07853890.2025.2478315. 40110683 10.1080/07853890.2025.2478315 PMC11926898

[35] Kim HS, Kim KH, Kim KH et al. Whole blood epstein-barr virus DNA load as a diagnostic and prognostic surrogate: extranodal natural killer/T-cell lymphoma. Leuk Lymphoma. 2009;50(5):757 – 63. 10.1080/10428190902803669 PMID: 19330658. 10.1080/10428190902803669 19330658

[36] Chen Y, Zheng X, Chen B, et al. The clinical significance of Epstein-Barr virus DNA in peripheral blood mononuclear cells in patients with non-Hodgkin lymphoma. Leuk Lymphoma. 2017;58(10):2349–55. 10.1080/10428194.2017.1300894. 28306367 10.1080/10428194.2017.1300894

[37] Wei W, Wu P, Li L, Zhang ZH. Effectiveness of pegaspargase, gemcitabine, and oxaliplatin (P-GEMOX) chemotherapy combined with radiotherapy in newly diagnosed, stage IE to IIE, nasal-type, extranodal natural killer/T-cell lymphoma. Hematology. 2017;22(6):320–9. 10.1080/10245332.2016.1264163. 27917702 10.1080/10245332.2016.1264163

[38] Mao J, Yin H, Wang L, Wu JZ, Xia Y, Zhu HY, Fan L, Li JY, Liang JH, Xu W. Prognostic value of 25-hydroxy vitamin D in extranodal NK/T cell lymphoma. Ann Hematol. 2021;100(2):445–53. 10.1007/s00277-020-04320-y. 33140135 10.1007/s00277-020-04320-y

[39] Sun Z, Wan W, Zhang X, et al. The clinical characteristics and prognostic factors of 410 patients with natural killer/T-cell lymphoma. J Cancer Res Clin Oncol. 2022;148(12):3449–59. 10.1007/s00432-022-04203-x. 35857124 10.1007/s00432-022-04203-x PMC11801057

[40] Kim SJ, Park S, Kang ES, Choi JY, Lim DH, Ko YH, Kim WS. Induction treatment with SMILE and consolidation with autologous stem cell transplantation for newly diagnosed stage IV extranodal natural killer/T-cell lymphoma patients. Ann Hematol. 2015;94(1):71–8. 10.1007/s00277-014-2171-4. 25082384 10.1007/s00277-014-2171-4

[41] Wang L, Wang H, Wang JH, et al. Post-treatment plasma EBV DNA positivity predicts early relapse and poor prognosis for patients with extranodal NK/T cell lymphoma in the era of asparaginase. Oncotarget. 2015;6(30):30317–26. 10.18632/oncotarget.4505. 26210287 10.18632/oncotarget.4505 PMC4745801

[42] Wang X, Zhang L, Liu X, et al. Efficacy and Safety of a Pegasparaginase-Based Chemotherapy Regimen vs an L-asparaginase-Based Chemotherapy Regimen for Newly Diagnosed Advanced Extranodal Natural Killer/T-Cell Lymphoma: A Randomized Clinical Trial. JAMA Oncol. 2022;8(7):1035–41. 10.1001/jamaoncol.2022.1968. 35708709 10.1001/jamaoncol.2022.1968 PMC9204617

[43] Alaggio R, Amador C, Anagnostopoulos I, et al. The 5th edition of the World Health Organization Classification of Haematolymphoid Tumours: Lymphoid Neoplasms. Leukemia. 2022;36(7):1720–48. 10.1038/s41375-022-01620-2. 35732829 10.1038/s41375-022-01620-2 PMC9214472

[44] Suzuki R, Yamaguchi M, Izutsu K, et al. Prospective measurement of Epstein-Barr virus-DNA in plasma and peripheral blood mononuclear cells of extranodal NK/T-cell lymphoma, nasal type. Blood. 2011;118(23):6018–22. 10.1182/blood-2011-05-354142. 21984805 10.1182/blood-2011-05-354142

